# Unraveling the Obesogenic Mechanism of Bisphenol A Through Network Toxicology and Molecular Docking: Identification of Key Molecular Targets

**DOI:** 10.3390/ijms262110647

**Published:** 2025-10-31

**Authors:** Ruiqiu Zhang, Manman Zhao, Hairuo Wen, Zhi Lin, Xiaobing Zhou

**Affiliations:** 1 National Institutes for Food and Drug Control, Chinese Academy of Medical-Sciences and Peking Union Medical College, Beijing 100730, China; 2 National Center for Safety Evaluation of Drugs, National Institutes for Food and Drug Control, Beijing 100176, China

**Keywords:** bisphenol A, network toxicology, obesity, molecular docking, lipid and atherosclerosis signaling pathway

## Abstract

This study integrates network toxicology with molecular docking technology to systematically elucidate the key molecular mechanisms and signaling pathways by which bisphenol A (BPA) induces obesity. By cross-referencing multiple databases—including the Comparative Toxicogenomics Database (CTD), SwissTarget prediction platform, and PharmMapper—potential BPA target genes were identified, yielding a total of 1326 candidate targets. Obesity-related genes were collected from GeneCards and OMIM databases, yielding 4570 disease-associated targets. Among these, 653 overlapping genes were identified as potential mediators linking BPA exposure to obesity. Protein interaction networks were constructed using STRING and Cytoscape, and the MCC algorithm identified five core hub genes: STAT3, MYC, TP53, IL6, and mTOR. Validation using random datasets demonstrated significant upregulation of these genes in the obesity group (*p* < 0.05), highlighting their potential central role in BPA-induced obesity effects. Functional enrichment analysis via GO and KEGG pathways indicated that BPA may promote obesity by interfering with endocrine signaling, activating lipid metabolism, and stimulating atherosclerosis pathways. Molecular docking analysis using CB-Dock2 confirmed strong binding affinity between BPA and core targets, providing structural evidence for their potential interactions. This study elucidates the potential biological mechanism by which BPA exacerbates obesity through endocrine disruption and metabolic reprogramming, employing a multidimensional approach encompassing cross-target analysis, pathway enrichment, and molecular interactions. It provides an innovative systems toxicology framework and empirical basis for assessing metabolic health risks induced by environmental pollutants.

## 1. Introduction

Obesity is a serious public health problem threatening the health of residents worldwide and in China, and it is a major risk factor for many chronic diseases [1]. Obesity is a state in which excessive fat, especially triglycerides, accumulates in the body due to excessive food intake or changes in the body’s metabolism. Research indicates that approximately 2.2 billion people worldwide are overweight, with about 712 million classified as obese. At present, more than 50% of adults and about 20% of school-age children in China are overweight or obese [2,3]. With the change of people’s lifestyles, bad eating habits represented by high-fat diets are becoming increasingly common, which is one of the main causes of obesity. Studies have shown that BPA is associated with generalized abdominal obesity [4], the concentration of BPA in adult urine is significantly correlated with obesity and overweight, and the difference is statistically significant [5,6,7].

Bisphenol A (BPA; C_15_H_16_O_2_) is a prototypical environmental endocrine disruptor exhibiting estrogenic activity. Commonly utilized as a raw material for plastics and epoxy resins, it finds extensive application in rubber goods, plastic products, food packaging liners, and printed circuit boards [8,9]. Hydroxyl groups in BPA enable migration from polymer matrices into environmental media. Release rates escalate significantly under high heat or acidic/alkaline conditions. Primary exposure routes include food packaging migration (>70% of daily intake), contaminated water, and dermal contact (e.g., receipts). Its lipophilic nature promotes adipose tissue bioaccumulation, amplifying long-term health risks [10]. BPA binds to a diverse array of receptors upon entering the body. These include classic steroid and thyroid hormone receptors (ERα/β, AR, TR, PR, RAR), as well as nuclear receptors involved in metabolism (PPARγ, RXR), immune receptors (TLRs, NLRs), the membrane-associated receptor GPR30, and orphan receptors such as the aryl hydrocarbon receptor (AhR) [11,12,13]. Previous research demonstrates that BPA’s binding affinity for hormone receptors constitutes a significant promoter of obesity development. Specifically, when BPA engages with ERα and ERβ, it induces aberrant activation of adipogenic regulatory pathways—thereby stimulating de novo adipocyte formation from precursor cells [12,14]. This combination leads to dysplasia of adipose tissue and causes obesity and metabolic disorders. Through this interaction, BPA can enhance lipid accumulation in adipocytes, stimulate precursor cells to differentiate into mature adipocytes, and thereby increase fat mass. Furthermore, the interaction between BPA and estrogen receptors can interfere with leptin and adiponectin signals [15,16,17], by regulating energy balance and insulin sensitivity, these hormones contribute to metabolic dysfunction and elevate susceptibility to weight gain and obesity.

However, current research predominantly examines isolated targets or pathways, limiting comprehensive analysis of BPA’s multifaceted toxicity mechanisms. This knowledge gap motivates Network Toxicology (NT)—an emerging discipline enabling systematic mapping of environmental pollutant toxicity. NT constructs multilayer “compound-target-disease” networks integrated with bioinformatics (e.g., STRING, Cytoscape) to identify critical hub genes and signaling pathways. Complementary molecular docking simulates ligand-receptor binding conformations, predicts binding affinity/structure-activity relationships, and provides computational assessment for experimental validation.

Based on the above challenges, this study proposes a multi-level research framework integrating network toxicology and molecular docking, aiming to systematically reveal the molecular mechanism of BPA-induced obesity. This study not only provides molecular evidence for the health risk assessment of BPA, but also lays a theoretical foundation for the development of intervention strategies targeting the metabolic-inflammatory pathway (such as STAT3 inhibitors and mTOR modulators), which has important scientific and social value.

## 2. Results

### 2.1. Procurement of Targets

**Figure 1:**
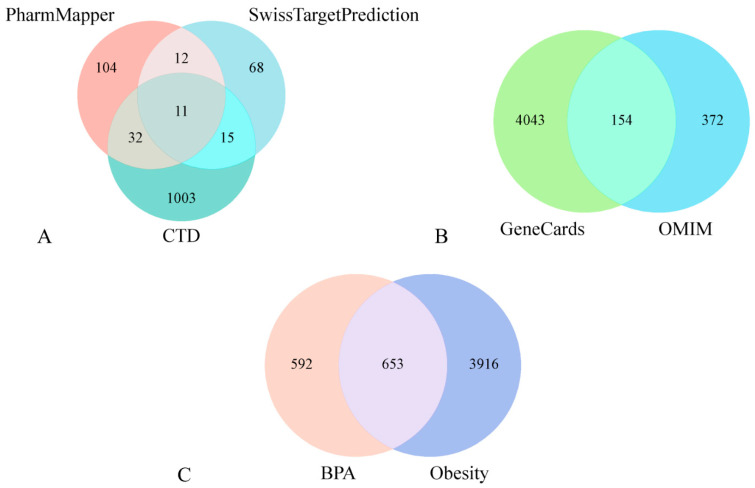
BPA target acquisition (**A**). Obesity target sourcing (**B**). Shared BPA-obesity targets (**C**).

Three databases (CTD, SwissTargetPrediction, PharmMapper) yielded 1326 potential BPA target genes (Figure 1A). GeneCards and OMIM provided 4197 and 526 obesity-related targets, respectively. Following duplicate removal, 4569 unique obesity targets were retained (Figure 1B). Intersection analysis identified 653 shared genes between disease and BPA targets (Figure 1C), representing core targets strongly linked to BPA-induced obesity.

### 2.2. PPI Network Analysis of Common Genes and Core Targets

**Figure 2:**
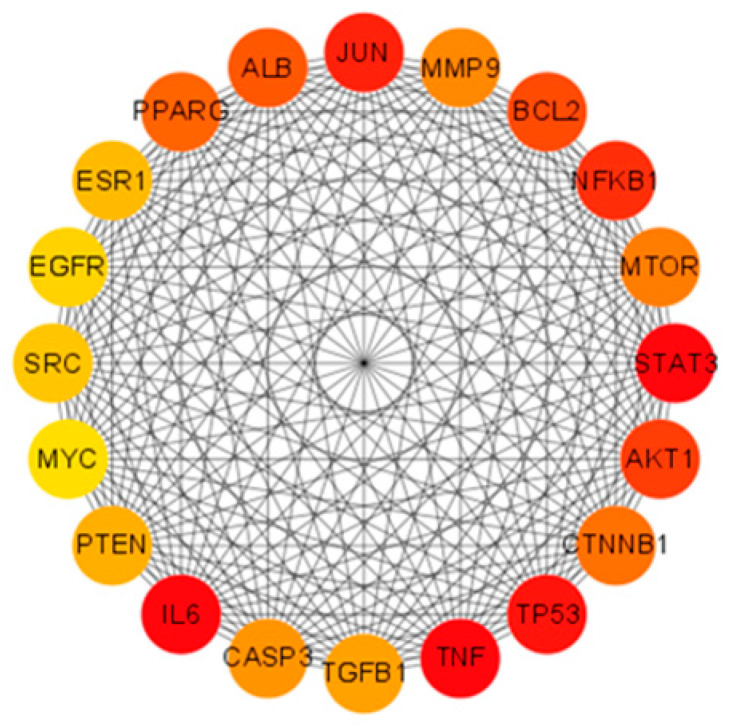
Core hub genes were systematically identified through application of the Matthews Correlation Coefficient (MCC) algorithm, a robust metric for assessing node significance in biological networks. The resultant network visualization illustrates complex functional relationships between these 20 key targets. Among them, STAT3, TNF, TP53, NFKB1, and IL6 achieved maximal MCC values, indicated by their prominent red-colored nodes in the diagram, reflecting their critical roles in mediating BPA-induced obesity mechanisms.

Utilizing the STRING database, a comprehensive protein–protein interaction (PPI) network was constructed for the 653 shared targets. This network comprised 644 interconnected nodes and 18,027 edges, yielding an average node degree of 56. The resultant network data, exported in TSV format from STRING, was subsequently imported into Cytoscape software for advanced topological analysis and visualization. To pinpoint pivotal genes within this complex network, we employed the CytoHubba plugin. Leveraging the Matthews Correlation Coefficient (MCC) algorithm—a methodologically rigorous scoring approach noted for its predictive accuracy—within CytoHubba, we systematically identified and visualized the top 20 core hub genes: PPARG, ALB, JUN, MMP9, BCL2, NFKB1, mTOR, STAT3, AKT1, CTNNB1, TP53, TNF, TGFB1, CASP3, IL6, PTEN, MYC, SRC, EGFR, and ESR1. These critical targets are graphically represented in Figure 2.

### 2.3. Core Gene Verification via Randomized Dataset Analysis

**Figure 3:**
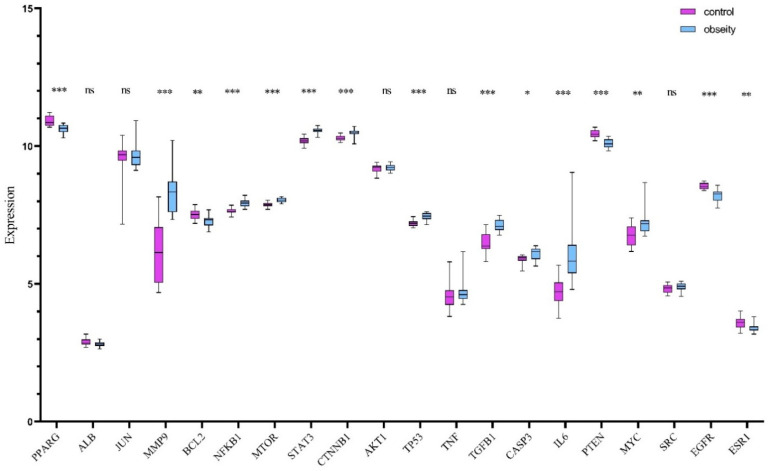
Differential expression profiles of 20 core genes across random data sets. Purple: control; Blue: obesity. * *p* < 0.05; ** *p* < 0.01; *** *p* < 0.001; ns: not significant.

To independently verify the expression patterns of core targets, the GSE59034 dataset was randomly selected for validation. This cohort comprises 16 samples from healthy individuals (control group) and 16 samples from clinically diagnosed obesity patients (disease group). Differential expression analysis of core genes between cohorts was conducted using Student’s *t*-test. Results demonstrated statistically significant dysregulation (*p* < 0.05) in the majority of core targets, with marked overexpression observed in the obesity group relative to healthy controls. These findings, illustrating consistent transcriptional upregulation in obesity pathogenesis, are comprehensively visualized in Figure 3.

### 2.4. Enrichment Analyses for GO Terms and KEGG Pathways

**Figure 4:**
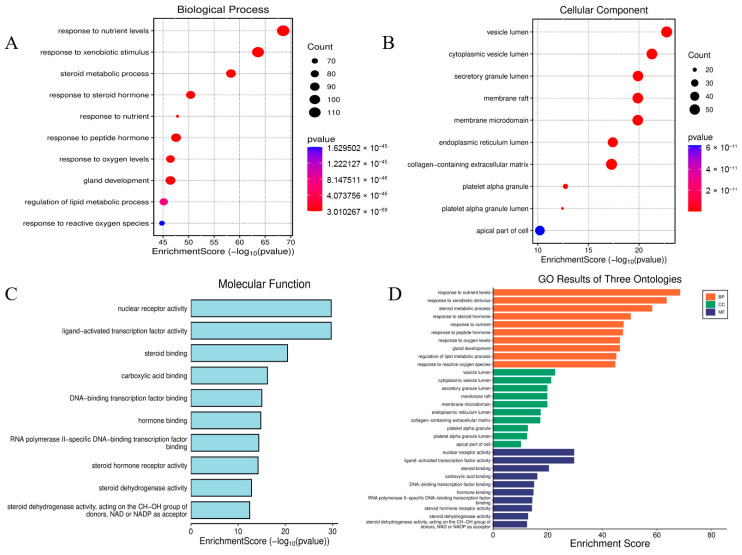
Go analysis diagram. GO-BP Enrichment: Cellular Stress Responses, Steroid Metabolism, and Nutrient/Hypoxia Adaptation (**A**). GO-CC Enrichment: Extracellular Matrix, Membrane Microdomains (Rafts) (**B**). GO-MF Enrichment: Receptor–Ligand Interactions and Cytokine Activity (**C**). Bar graph of GO analysis (**D**).

**Figure 5:**
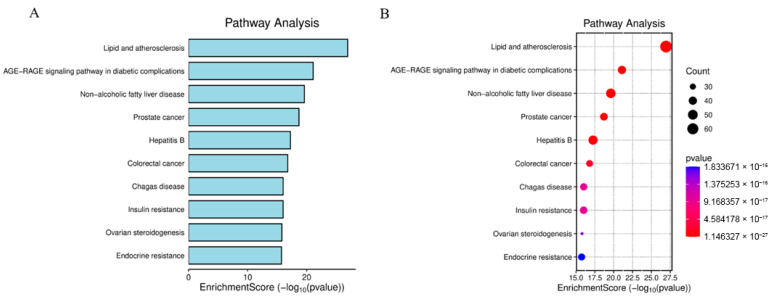
Bar Chart of Top 10 KEGG Pathways (**A**). Bubble Plot of Key KEGG Enrichment (**B**). Primary pathways: Diabetic complications, Lipid metabolism & atherosclerosis, Endocrine resistance, Steroid hormone biosynthesis, TNF signaling.

To elucidate the pathophysiological mechanisms underlying BPA-exacerbated obesity, integrated functional enrichment analyses were performed through Gene Ontology (GO) annotation and Kyoto Encyclopedia of Genes and Genomes (KEGG) pathway mapping. Focusing on the 653 intersecting targets, these computational investigations were executed using the “clusterProfiler” package (https://www.bioinformatics.com.cn/?p=1 accessed on 27 October 2025) within the R statistical environment. The ten most significantly enriched Biological Process (GO-BP) terms, prioritized by ascending adjusted *p*-values, revealed fundamental involvement in adaptive cellular responses to environmental stressors, steroid hormone biosynthesis and metabolism, homeostatic regulation of nutrient fluctuations, and hypoxia-induced cellular adaptation mechanisms. For Cellular Component (GO-CC) annotations, top-ranked terms highlighted critical structural domains including collagen-enriched extracellular matrices, membrane microdomains (lipid rafts), endoplasmic reticulum luminal compartments, and vesicular lumen architectures. Molecular Function (GO-MF) analysis demonstrated essential roles in receptor-ligand binding specificity, cytokine receptor engagement, cytokine-mediated signaling cascades, and growth factor regulatory activities, with comprehensive visualization provided in Figure 4. Concurrent KEGG pathway interrogation identified 177 significantly dysregulated pathways (adjusted *p* < 0.05), predominantly clustered in diabetic complication cascades, lipid metabolism dysregulation and atherogenesis, endocrine resistance mechanisms, steroid hormone biosynthesis, TNF and MAPK signal transduction networks, and AGE-RAGE axis activation in oncogenic contexts. The hierarchical profiling of the top 10 KEGG pathways is systematically depicted through dual graphical representations (bar and bubble charts) in Figure 5.

### 2.5. Molecular Docking of BPA with Obesity Core Targets

**Figure 6:**
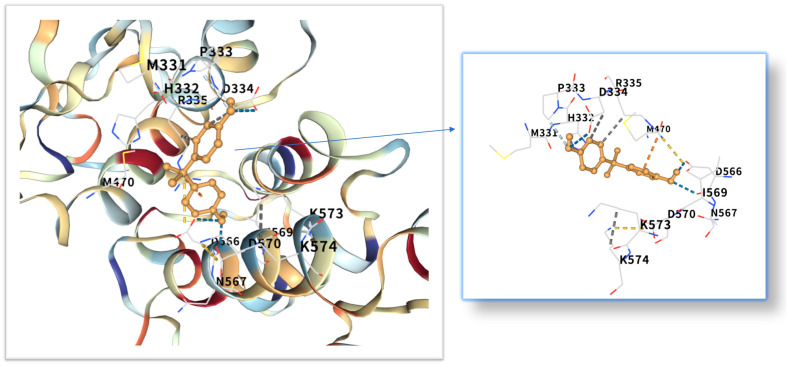
Three-dimensional molecular docking model of BPA (shown as yellow sticks) bound to the STAT3 protein (PDB: 6NJS, depicted as a cyan cartoon).

**Figure 7:**
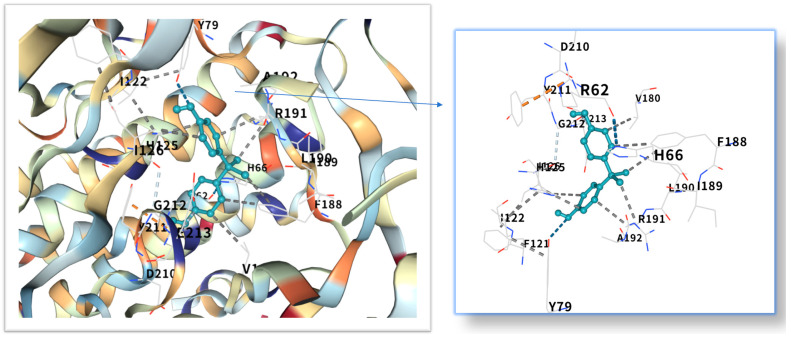
Three-dimensional molecular docking model of BPA (shown as green sticks) bound to the IL-6 protein (PDB: 8XWY, depicted as a cyan cartoon).

**Figure 8:**
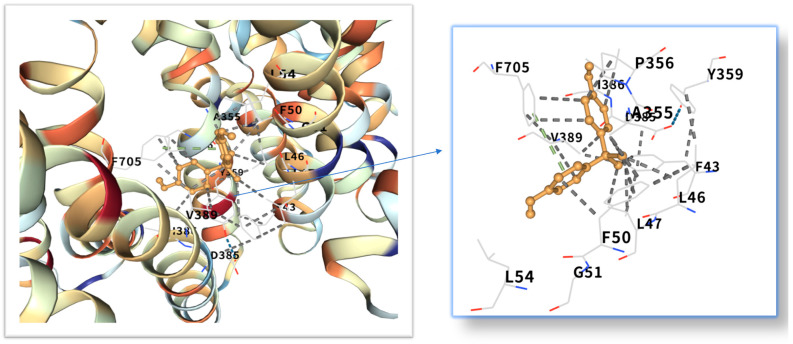
Three-dimensional molecular docking model of BPA (shown as yellow sticks) bound to the mTOR protein (PDB: 8WR3, depicted as a cyan cartoon).

**Figure 9:**
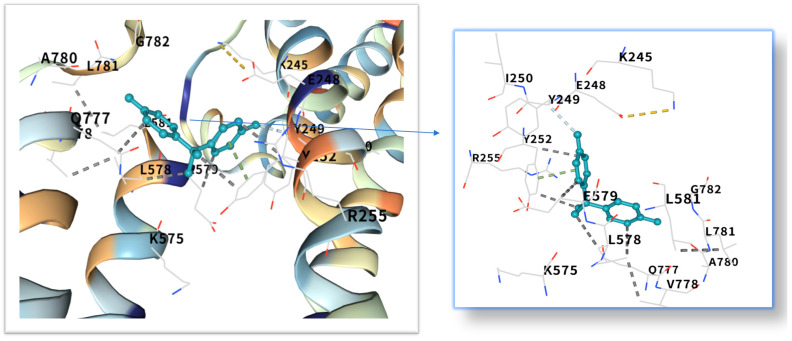
Three-dimensional molecular docking model of BPA (shown as green sticks) bound to the MYC protein (PDB: 1NKP, depicted as a cyan cartoon).

**Figure 10:**
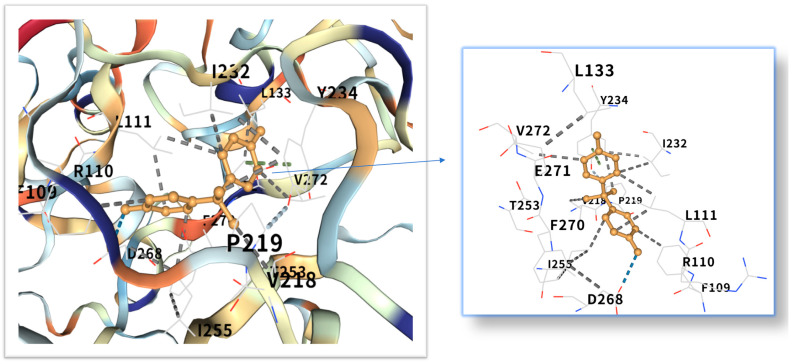
Three-dimensional molecular docking model of BPA (shown as yellow sticks) bound to the TP53 protein (PDB: 9CHT, depicted as a cyan cartoon).

The five highest-ranking core targets—STAT3, IL-6, mTOR, MYC, and TP53—were prioritized for subsequent molecular docking investigations. This prioritization was based on their attainment of the highest Matthews Correlation Coefficient (MCC) scores derived from the preceding CytoHubba analysis, a result indicative of their central topological importance within the protein–protein interaction (PPI) network. To probe the potential intermolecular recognition events, molecular docking analyses were systematically performed to characterize the binding interactions between the ligand Bisphenol A (BPA) and each of these five key hub targets: STAT3 (utilizing PDB structure 6NJS), mTOR (PDB: 8WR3), TP53 (PDB: 9CHT), IL-6 (PDB: 8XWY), and MYC (PDB: 1NKP). The PDB structures for STAT3 (6NJS), mTOR (8WR3), TP53 (9CHT), IL-6 (8XWY), and MYC (1NKP) were selected based on high resolution, structural completeness, and biological relevance to ligand binding. These structures represent functional conformations commonly used in molecular docking studies for these targets. The computational docking results generated by the CB-Dock2 platform revealed calculated binding energies for the BPA-target complexes spanning a range from −6.1 to −8.7 kcal/mol. The results are shown in Table 1. Within the context of molecular docking energetics, binding free energy values less than zero (<0 kcal/mol) generally signify favorable binding activity, while values falling below −5.0 kcal/mol are typically interpreted as indicative of strong binding affinity, with progressively lower (more negative) values corresponding to increasingly stable interactions. These computational findings clearly demonstrated robust binding affinities between BPA and all five prioritized core targets. The observed favorable binding energies imply a spontaneous binding process, thereby suggesting that these specific targets play a potentially critical mechanistic role in mediating the molecular pathways underlying BPA-induced obesity. The detailed three-dimensional (3D) binding poses and interaction patterns for each BPA-core target complex, as computed by CB-Dock2, are comprehensively visualized and presented in Figure 6, Figure 7, Figure 8, Figure 9 and Figure 10. The enlarged image shows the docking diagram with the receptor framework hidden, allowing for a clearer visualization of the docking content and precise positioning.

## 3. Discussion

Obesity is a major global health problem, influenced by various factors, including environmental conditions. It is related to insulin resistance, inflammation and oxidative stress [18]. Researchers are increasingly concerned about the health risks of BPA [19], which is known for its toxicity, endocrine disruption and potential carcinogenic effects [20,21,22,23]. Even at low concentrations, endocrine disrupting chemicals (EDCs) can act by interfering with hormone-receptor interactions [24], affecting the secretion, metabolism, transport, release and elimination of hormones. They can bind to membrane, cytoplasmic or nuclear receptors, including ERα, ERβ, androgen receptor, aromatic hydrocarbon receptor, progesterone receptor, glucocorticoid receptor and progeane-X [9,25]. However, its impact on obesity remains unclear and further research is needed. The STRING and Cytoscape data were used to construct a PPI network, and the MCC algorithm was applied to identify five key core targets: STAT3, IL-6, mTOR, MYC, and TP53. STAT3, as a core member of the JAK-STAT signaling pathway, its phosphorylation can promote adipocyte differentiation and the secretion of inflammatory factors (such as IL-6, TNF-α) [26,27]. Although mTOR and MYC were not the top-ranked candidate targets in the CytoHubba analysis in the McC-based hub list, their expressions showed significant differences in the comparison between obese patients and normal individuals in the random validation dataset. Moreover, because they are recognized as the main regulatory factors of lipid metabolism and adipogenesis, and are empirically associated with BPA-induced metabolism [28], these two factors have been used for further verification of molecular docking. Molecular docking shows that the binding energy of BPA to STAT3 is −6.1 kcal/mol, and the binding is relatively stable. Previous studies have reported that the combination of BPA and STAT3 may induce diabetic cardiomyopathy [29]. More and more evidence indicates that BPA exposure is associated with a series of metabolic disorders, including obesity, neurodegenerative diseases and metabolic syndrome [30,31]. This binding mode is highly similar to the dimerization of STAT3 induced by IL6, suggesting that BPA may continuously activate STAT3 signaling through “quasi-cytokine” action [32]. Animal experiments further support this mechanism: BPA exposure (50 μg/kg/day) can increase the phosphorylation level of STAT3 in mouse adipose tissue by 2.3 times and significantly up-regulate the expression of adipogenic-related genes (PPARγ, C/EBPα) [33,34]. Furthermore, STAT3 inhibits the phosphorylation of insulin receptor substrates (IRS) by up-regulating SOCS3, exacerbates insulin resistance, and forms a vicious cycle of metabolism and inflammation [35,36]. IL6, as a classic pro-inflammatory cytokine, plays a dual role in BPA-induced obesity [37,38]. Its binding energy with bisphenol A is −7.1 kcal/mol. The verification of the random dataset in this study showed that the level of IL6 mRNA in the obese group was higher than that in the control group (*p* < 0.01). Furthermore, IL6 inhibits insulin signal transduction by activating the JNK/AP-1 pathway, resulting in a 30% decrease in insulin sensitivity of adipocytes [39,40]. Clinical data show that high IL6 levels are positively correlated with visceral fat area (VFA) and serum triglyceride (TG) concentrations [41], it is suggested that IL6 plays a central role in BPA-induced metabolic disorders. Building upon the mechanistic insights from Yang et al.’s investigation, their research demonstrated that IL-6 may serve as a pivotal molecular mediator through which polychlorinated biphenyl (PCB) exposure disrupts breast cancer pathogenesis [42]. These findings critically underscore that the synergistic interplay between inflammatory mediators (notably IL-6) and environmental toxicants represents a significant oncogenic risk factor—suggesting that pollutant-induced dysregulation of cytokine signaling networks may establish a permissive microenvironment for tumor initiation and progression. This evidence positions IL-6 not merely as a biomarker, but as a central mechanistic node in environmental carcinogenesis, warranting prioritized investigation into cytokine-pollutant crosstalk for cancer prevention strategies.

As a core regulatory factor of energy metabolism, the abnormal activity of mTOR is closely related to lipid droplet accumulation [43]. This study found that the binding energy of BPA to mTOR is −8.7 kcal/mol, and its binding stability is higher than that of several other targets. As a proto-oncogene, the mechanism by which MYC participates in lipid metabolism has gradually been revealed in recent years [44]. Relevant studies have shown that BPA exposure can increase the level of MYC mRNA in adipocytes by 1.8 times [45]. The binding energy of MYC and bisphenol A is −7.3 kcal/mol. Inhibition of MYC activity can reduce the triglyceride content of adipocytes by 40%, suggesting that it plays a key role in BPA—induced lipid accumulation [28]. While TP53 is classically recognized for its canonical roles in DNA repair and tumor suppression, emerging evidence indicates its metabolic involvement in obesity pathogenesis [46]. Molecular docking revealed potent binding between TP53 and bisphenol A (binding energy: −8.6 kcal/mol), suggesting direct biological relevance. Mechanistically, studies demonstrate that BPA activates TP53 through mitochondrial ROS accumulation, subsequently upregulating p21 and PUMA expression [47]. The p21 protein induces senescence in adipocyte precursor cells, impairing their differentiation into mature adipocytes, while PUMA triggers mitochondrial pathway-mediated apoptosis in mature adipocytes. This regulatory duality aligns with clinical observations: visceral adipose tissue from obese patients exhibits a 15% higher prevalence of TP53-positive cells compared to healthy controls, showing significant positive correlation with adipocyte apoptosis markers [48]. Collectively, these findings position TP53 as a multifunctional regulator of lipid homeostasis, orchestrating both cellular senescence and apoptotic pathways in adipose biology.

Subsequently, we conducted GO and KEGG enrichment analyses on the crossover genes, and the significant enrichment pathways were consistent with the potential impact of bisphenol A as an EDC on bone health. KEGG enrichment analysis was the first to link the “lipid and atherosclerosis” pathway with BPA-induced obesity, revealing a potential bridge between environmental pollutants and cardiovascular complications. As an EDC, bisphenol A can disrupt the endocrine regulation of the body, especially affecting insulin sensitivity [49], EDC has a negative impact on endogenous hormones, and subsequently affects the release of cytokines and growth factors, thereby influencing the occurrence and progression of obesity [50].

Molecular docking analysis revealed that these five core target proteins played a key role in bisphenol A-induced obesity. Bisphenol A forms stable interactions with the binding pockets of the above five core proteins, with binding energies ranging from −6.1 to −8.7 kcal/mol, demonstrating strong docking affinity. While molecular docking provided initial insights into BPA-target binding, future studies could leverage cryo-electron microscopy (cryo-EM) [51] to resolve high-resolution structures of BPA-bound complexes (e.g., BPA-STAT3 or BPA-mTOR). Besides, the methods of DEMO-EM [52] and EMBuild [53] can automatically construct multi-chain protein complex models from medium-resolution cryogenic maps, and their accuracy is comparable to that of the ab initio modeling method based on cryogenic electron microscopy. Emerging AI-driven tools (e.g., ModelAngelo [54], DiffModeler [55]) offer unprecedented capabilities to model ligand-induced conformational changes at near-atomic resolution. Such approaches would validate our docking predictions and reveal mechanistic details of BPA’s allosteric effects on key targets.

While this study provides a comprehensive in silico framework for elucidating BPA’s obesogenic mechanisms, it is important to acknowledge its primary limitation: the lack of in vivo or in vitro experimental validation, the following limitations still exist: (1) The main limitation is the lack of in vivo experiments to evaluate the impact of bisphenol A on obesity, which will verify core targets and effector pathways and promote the development of prevention and treatment strategies. The integration of such experimental data will be crucial to fully confirm the causal relationships inferred from our computational models. (2) Obesity is highly heterogeneous, and different subtypes (such as metabolic obesity vs. inflammatory obesity) may have different responses to BPA exposure. Future research needs to combine the single-cell sequencing data of clinical samples to analyze the specific effects of BPA on different adipocyte subsets. In the actual environment, BPA often coexists with pollutants such as bisphenol S (BPS) and phthalates. The synergistic or antagonistic effects of these compounds may significantly alter the toxic pathways and need to be systematically evaluated through in vitro combined exposure experiments. Most of the existing data are based on high-dose exposure models, while the long-term effects of environment-related low-dose (ng/mL level) still require long-term tracking studies. Quantifying the impact of EDC mixtures on obesity and improving the assessment of EDC exposure will enable more powerful inferences to be made from epidemiological studies than currently available.

## 4. Methods

### 4.1. Collection of Bisphenol A Targets

The chemical structure and SMILES notation of Bisphenol A (BPA) were acquired through PubChem queries. Potential targets of BPA were then systematically retrieved from three specialized databases: the Comparative Toxicology Genomics Database (CTD), SwissTargetPrediction, and PharmMapper. To ensure uniformity, all target identifiers were standardized to official gene symbols via UniProt’s ID mapping service, which consolidated entries from the aforementioned sources. Automated cross-referencing eliminated redundant targets, while protein interactions were rigorously filtered by retaining only high-confidence associations (STRING score > 0.7). This consolidated dataset, comprising merged and filtered targets, ultimately constituted the BPA target library.

### 4.2. Obesity Target Identification

Potential therapeutic targets for obesity were identified by querying “obesity” in GeneCards and OMIM databases. To ensure comprehensive coverage, retrieved targets from both repositories were consolidated into a unified obesity target dataset. The inclusion criteria encompassed: GeneCards relevance scores ≥ 1 (indicating above-average disease association) and OMIM entries annotated as pathogenic.

### 4.3. Visualization of “BPA-Obesity” Target Interactions

The Bisphenol A (BPA) targets and obesity-associated targets were mapped in a Venn diagram using the “VennDiagram” package in R (https://www.bioinformatics.com.cn/plot_basic_proportional_2_or_3_venn_diagram_028, accessed on 27 October 2025). This enabled systematic identification of cross-targets between the two sets. Functionally annotated details for each gene subset within the diagram are cataloged in Supplementary Table S1.

### 4.4. Protein–Protein Interaction Network Construction

Shared targets between Bisphenol A and obesity were input into the STRING database to generate a protein–protein interaction (PPI) network. Analysis parameters specified: Homo sapiens as the species and a minimum interaction confidence score > 0.7 (high reliability). The resultant TSV-formatted interaction file was imported into Cytoscape v3.9.1, where topological analysis using the cytohubba plugin identified hub proteins based on maximal clique centrality (MCC) scoring.

### 4.5. External Validation Using Transcriptomic Data

The gene expression matrix was acquired from the Gene Expression Omnibus (GEO) database using the search terms “obesity” and “Homo sapiens”. The GSE59034 dataset was prioritized for analysis, containing demographically matched cohorts: (1) 16 healthy controls (age: 45 ± 6 years; BMI: 22.3 ± 1.8 kg/m^2^), (2) 16 obese patients (age: 47 ± 5 years; BMI: 32.1 ± 3.2 kg/m^2^). Differential expression analysis of hub targets between groups was performed via Student’s *t*-test (*p* < 0.05 deemed significant), with results visualized in boxplots annotating statistical outliers.

### 4.6. Gene Ontology (GO) and Kyoto Encyclopedia of Genes and Genomes (KEGG) Enrichment Analyses

To delineate the mechanism of BPA-induced obesity, intersected targets were converted to Entrez IDs via the org.Hs.eg.db R package (https://www.uniprot.org/ accessed on 27 October 2025) [56]. These curated identifiers underwent functional enrichment analysis implemented in clusterProfiler, with GO term annotation and KEGG pathway mapping performed concurrently. Significantly enriched terms (*p*-value & q-value < 0.05) were filtered, yielding the top 10 GO processes and KEGG pathways ranked by ascending q-value (FDR-corrected significance). Result visualization integrated enrichplot and ggplot2 for multidimensional interpretation.

### 4.7. Molecular Docking Validation of BPA-Hub Target Interactions

Molecular docking was employed to characterize binding mechanisms between Bisphenol A (BPA) and obesity-associated hub targets, predicting binding modes and affinity energies through computational simulations. Within the context of molecular docking energetics, binding free energy values less than zero (<0 kcal/mol) generally signify favorable binding activity, while values falling below −5.0 kcal/mol are typically interpreted as indicative of strong binding affinity, with progressively lower (more negative) values corresponding to increasingly stable interactions [56,57,58]. Protein structures (receptors) and the BPA ligand were prepared as follows: (1) BPA’s isomeric SMILES was curated from PubChem, (2) Hub target PDB files were acquired via UniProt IDs using PDB’s advanced search, and (3) The CB-Dock2 platform automates ligand/protein preprocessing, including structure optimization (energy minimization, hydrogen atom addition) and solvent shell removal (explicit water molecule elimination) [59]. Regarding docking parameters and settings, we did not customize any parameters and performed automatic blind docking. Docking simulations identified optimal binding sites based on minimal Vina scores (≤−6.0 kcal/mol indicating strong binding). Docking validation was performed by re-docking the native ligands extracted from each PDB structure, achieving RMSD values < 2.0 Å, confirming the reliability of the docking protocol. During docking, ligand flexibility was handled by allowing full torsional freedom for all rotatable bonds of BPA, whereas receptor side chains within 5 Å of the binding site were kept flexible using the CB-Dock2 default settings. Binding affinities were estimated with the Vina scoring function, expressed as binding free energy (ΔG, kcal/mol).

### 4.8. Data Sources

All databases were accessed between January and June 2025. The specific version is shown in Table 2.

## 5. Conclusions

Through an integrated network toxicology and molecular docking approach, this study systematically deciphers the molecular pathogenesis of Bisphenol A—induced obesity, demonstrating its tripartite mechanism: endocrine signal disruption via receptor interactions, metabolic pathway dysregulation (including steroid biosynthesis and MAPK signaling), and high-affinity binding to core targets. Our multi-database analysis identified 1326 potential BPA targets and 4570 obesity-associated genes, intersecting at 653 shared targets that define the BPA-obesity molecular interface. Protein–protein interaction (PPI) network topology and Matthews Correlation Coefficient (MCC) scoring prioritized 20 hub genes—notably STAT3, MYC, TP53, IL-6, and mTOR—whose central network positions designate them as master regulators of obesogenesis. Critically, molecular docking validated strong binding affinities (binding energies: −6.1 to −8.7 kcal/mol) between BPA and top-ranked targets (STAT3, IL-6, mTOR, MYC, TP53), confirming their mechanistic involvement. Pathway enrichment revealed BPA’s coordinated activation of endocrine-disrupting pathways (AGE-RAGE, TNF signaling) and metabolic cascades, with the lipid and atherosclerosis pathway emerging as a novel contributor wherein BPA exacerbates obesity comorbidities through oxidative stress induction and lipid metabolism dysregulation. These findings establish three key conclusions: (1) BPA’s multi-target action underpins its obesogenic potency, (2) STAT3 and mTOR represent viable therapeutic targets, and (3) the lipid-atherosclerosis link reveals cardiovascular comorbidity risks—collectively necessitating enhanced regulation of BPA-containing products and accelerated development of molecular interventions. Future research should expand this integrated framework to assess multi-pollutant synergistic effects, validate core targets in disease models, and develop structure-based inhibitors, thereby translating mechanistic insights into evidence-based environmental health policies.

## Figures and Tables

**Table 1:** The result of Molecular docking.

Target	PDB ID	Binding Energy (kcal/mol)	Key Interacting Residues
STAT3	6NJS	−6.1	ALA250 CYS251 ILE252 GLY253 PRO256
IL-6	8XWY	−7.1	PHE68 HIS72 LEU83 GLY84 GLU85
mTOR	8WR3	−8.7	PHE43 LEU46 LEU47 PHE50 GLY51
MYC	1NKP	−7.3	GLU957 GLN958 ILE961 GLU964 ASP965
TP53	9CHT	−8.6	LEU413 GLY414 GLU415 GLU416 ARG418

**Table 2:** Data source and access link.

Database Name	Access Address
CTD	https://ctdbase.org/
SwissTargetPredition	http://swisstargetprediction.ch/
PharmMapper	https://www.lilab-ecust.cn/pharmmapper/ (accessed on 27 October 2025)
GeneCards	https://www.genecards.org/
OMIM	https://www.omim.org/
UniProt	https://www.uniprot.org/
STRING	https://cn.string-db.org/
GEO	https://www.ncbi.nlm.nih.gov/gds (accessed on 27 October 2025)
CB-DOCK2	CB-Dock2: An accurate protein-ligand blind docking tool

## Data Availability

The original contributions presented in this study are included in the article/Supplementary Materials. Further inquiries can be directed to the corresponding author.

## Supplementary Materials

The following supporting information can be downloaded at: https://www.mdpi.com/article/10.3390/ijms262110647/s1.
